# The negative relationship between brain-age gap and psychological resilience defines the age-related neurocognitive status in older people

**DOI:** 10.1007/s11357-025-01515-x

**Published:** 2025-01-28

**Authors:** Yue Gu, Nichol M. L. Wong, Chetwyn C. H. Chan, Jingsong Wu, Tatia M. C. Lee

**Affiliations:** 1 State Key Laboratory of Brain and Cognitive Sciences, The University of Hong Kong, Hong Kong, China; 2 Laboratory of Neuropsychology and Human Neuroscience, The University of Hong Kong, Hong Kong, China; 3 Department of Psychology, The Education University of Hong Kong, Hong Kong, China; 4 Centre for Psychosocial Health, The Education University of Hong Kong, Hong Kong, China; 5 College of Rehabilitation Medicine, Fujian University of Traditional Chinese Medicine, Fuzhou, China; 6 The Academy of Rehabilitation Industry, Fujian University of Traditional Chinese Medicine, Fuzhou, China

**Keywords:** Brain-age gap, Resilience, Ageing, Neurocognitive status

## Abstract

Biological brain age is a brain-predicted age using machine learning to indicate brain health and its associated conditions. The presence of an older predicted brain age relative to the actual chronological age is indicative of accelerated aging processes. Consequently, the disparity between the brain’s chronological age and its predicted age (brain-age gap) and the factors influencing this disparity provide critical insights into cerebral health dynamics during aging. In this study, we employed a Lasso regression model and analyzed multimodal imaging data from 124 participants aged 53 to 76 to formulate and predict brain age. Additionally, we conducted partial correlation analyses to explore the complex relationship between the brain-age gap and network metrics, cognitive assessments, and emotional evaluations, while controlling for chronological age, gender, and education. Our findings highlight psychological resilience as a significant mitigating factor against premature brain aging. It is established that psychological resilience significantly influences the modulation of the brain-age gap. Moreover, psychological resilience and the brain-age gap exhibit a high accuracy (above 0.72) in segregating Montreal Cognitive Assessment score–based cohorts. This observation underscores significant insight into the potential of utilizing the brain-age gap as a diagnostic tool for the early detection of accelerated aging. It advocates for the timely application of interventions, including the development of programs aimed at bolstering psychological resilience.

**Supplementary Information:**

The online version contains supplementary material available at 10.1007/s11357-025-01515-x.

## Introduction

Under the influence of various factors and health status, the complex biological process of ageing unfolds at varying rates, leading to the discrepancy of brain biological and chronological ages [1]. Following the established biogerontological model of determining the disparity between the chronological and biological age [2], individuals with older predicted-brain age than chronological age might imply accelerated brain ageing that predisposes to brain diseases. This deviation between the predicted-brain age and chronological age is referred to as the “brain-age gap” [3]. There are associations between individual brain-age gaps and adverse outcomes such as mortality [4] and neurodegenerative disorders [5–7].

Researchers strive to identify objective measures of brain biological age [8, 9] that can better understand the potential risk of neurocognitive degenerative conditions than the chronological age alone. In this regard, given that ageing is associated with diverse structural and functional changes within the brain [10–13], structural and functional magnetic resonance imaging (MRI) data together with diffusion tensor imaging (DTI) findings offer detailed insights into the structural and functional changes occurring in the ageing brain [14–17]. These imaging techniques facilitate the derivation of the brain’s biological age, termed brain-predicted age, potentially serving as a reliable biomarker [18–21]. Utilizing structural or functional neuroimaging data, predictive models that leverage machine learning techniques are trained on datasets to estimate brain age by comparing it to chronological age, by extracting various features [18, 20–27]. These models usually used the structural or/and stationary functional brain network as features. Besides, the dynamic functional brain network is also valuable to consider, which facilitates the observation of details that are [28, 29], and may also provide potential sensitive clinical biomarkers [30, 31]. The prediction of brain age through the integration of multimodal neuroimaging features remains limited, thereby potentially overlooking the cumulative importance of various data modalities in the prediction process.

The ageing process often brings forth a myriad of challenges, including chronic illness and disability [32, 33]. Within this context, psychological resilience emerges as a crucial factor in an individual’s ability to adapt to and recover from stress, adversity, or challenges [34]. Significantly, resilience has been linked to successful aging, possessing an impact that rivals the importance of physical health [35]. It has significant association with successful aging, with effects comparable in size to that for physical health [36]. Previous studies have identified that effective measures to promote resilience are likely to have a positive effect on older people’s lifespan [37, 38]. The brain-age gap quantifies individuals’ brain health as deviation from a normative brain ageing trajectory. A higher brain-age gap is associated with lower life satisfaction and less favorable health characteristics in ageing, potentially due to diminished resilience [35]. Given the resilience can be improved through targeted interventions [32, 39], exploring the relationship between the brain-age gap and resilience may provide valuable insights into risk factors, guide the development of interventions, and facilitate outcome measurement in aging populations.

In our study, we developed a deep learning–based brain age prediction model using multimodal imaging data from 124 participants aged 53–76 years. The Lasso regression [40] was applied to multimodal imaging data to facilitate variable selection and regularization for predicting the brain age. The model using multimodal neuroimaging features ascertains the discrepancy between chronological and predicted brain age, termed the brain-age gap. We further investigate the association of the brain-age gap with brain network metrics. Through partial correlation analyses that account for age, gender, and education, we examined the complex relationship between the brain-age gap, network metrics, and cognitive and emotional assessments. The Canonical Correlation Analysis (CCA) further explored the relationship between neurocognitive age-related characteristics and widely used cognitive performance measures. Moreover, we stratified the participants into two groups based on their Montreal Cognitive Assessment (MOCA) scores to verify any significant between-group differences in neurocognition and brain-age gap. Last but not least, we conducted k-means clustering to assess the accuracy of distinguishing MOCA-related groups based on both brain-age gap and resilience, given that, according to the literature, resilience is a significant protective factor of brain health.

## Methods

### Participants

Demographic variables were assessed at the time of the scan. The data were obtained from a cohort of 124 right-handed older adults who underwent a comprehensive neuropsychological assessment, including the Connor-Davidson Resilience Scale [41], Montreal Cognitive Assessment (MOCA) [42], Pittsburgh Sleep Quality Index (PSQI) [43], Instrumental Activities of Daily Living (IADL) [44], Alzheimer’s Disease 8-item Informant Interview (AD8) [45], Geriatric Depression Scale (GDS) [46], University of California Los Angeles Loneliness Scale (UCLA_LS) [47], Perceived Stress Scale (PSS) [48], and Lubben Social Network Scale (LSNS) [49]. Inclusion criteria were applied as follows: absence of severe sensory or motor impairments that could interfere with cognitive testing; complete availability of imageing data (with 11 participants removed due to missing data); head motion within 3 mm/degree of tolerance (with five participants excluded); absence of a diagnosis and/or symptoms of depression, indicated by a GDS score greater than 5/15, and a GDS score no higher than 5 (resulting in the exclusion of 15 participants).

The final sample consisted of 93 participants, with a mean age of 64.41 years (SD = 5.66 years, range = 53–76 years), including 33 males. This study received approval from the institutional review board of the Affiliated Rehabilitation Hospital, Fujian University of Traditional Chinese Medicine, and all participants provided written informed consent. A summary of all data can be found in Table 1.

**Table 1:** Participant demographic and performance characteristics

	Total (mean ± SD)
**Age** (*n* = 93)	64.41 ± 5.66
**Gender** (*n* = 93)	33 M
**Resilience** (*n* = 70)	65.4 ± 17.29
**MOCA** (*n* = 93)	21.78 ± 3.24 (14–28)
**PSQI** (*n* = 92)	6.20 ± 3.50 (1–18)
**IADL** (*n* = 93)	22.70 ± 0.59 (21–23)
**AD8** (*n* = 93)	1.51 ± 1.56 (0–6)
**GDS** (*n* = 93)	2.27 ± 1.46 (0–5)
**UCLA_LS** (*n* = 93)	31.28 ± 8.56 (20–58)
**PSS** (*n* = 88)	24.10 ± 7.40 (11–47)
**LSNS** (*n* = 73)	17.59 ± 4.86 (7–29)

For each item, *n* represents the number of participants, and the range within parentheses after mean ± SD represents the range of values for the corresponding scale

*SD* standard deviation, *M* male, *Resilience* psychological resilience by Connor-Davidson Resilience Scale, *MOCA* Montreal Cognitive Assessment, *PSQI* Pittsburgh Sleep Quality Index, *IADL* Instrumental Activities of Daily Living, *AD8* Alzheimer’s Disease 8-item Informant Interview, *GDS* Geriatric Depression Scale, *UCLA_LS* University of California Los Angeles Loneliness Scale, *PSS* Perceived Stress Scale, *LSNS* Lubben Social Network Scale

### Data acquisition and preprocessing

All 93 participants underwent scanning on a Prisma Simens 3.0 scanner. Before the scanning, participants were instructed to rest and relax to minimize the potential head motion and overthinking, especially in the resting-state period. Resting-state functional MRI data was acquired using an echo-planar imaging (EPI) sequence with the following parameters: field of view (FOV) = 224 mm, repetition time (TR)/echo time (TE) = 2000/30 ms, number of slices = 37, total volume = 270, slice thickness = 3.5 mm, voxel size = 3.5 × 3.5 × 3.5 mm^3^, and flip angle (FA) = 90°. High-resolution T1-weighted anatomical images were obtained using a magnetization-prepared rapid gradient echo (MPRAGE) sequence, with FOV = 256 mm, TR/TE = 2530/2.51 ms, slice thickness = 1.0 mm, slices per slab = 192, voxel size = 1.0 × 1.0 × 1.0 mm^3^, and FA = 7°.

The acquired images underwent preprocessing using the Data Processing & Analysis of Brain Imaging (DPABI) (http://rfmri.org/dpabi [50]) and SPM12 (https://www.fil.ion.ucl.ac.uk/spm/). For each participant, the preprocessing steps included discarding the initial five volumes, motion correction, segment gray matter (GM), white matter (WM), and cerebrospinal fluid (CSF) tissue maps from individual T1-weighted images, normalization to the Montreal Neurological Institute (MNI) template, resampling to 3.0 × 3.0 × 3.0 mm^3^, regression of nuisance variables (Friston’s 24 head motion parameters, global signal, white matter, and cerebrospinal fluid signals), and band-pass filtering (0.01–0.1 Hz). After the normalization of GM, WM, and CSF, the resulting images were resampled to 3 ${\text{mm}}^{3}$ and smoothed using a 6-mm full width at half maximum Gaussian kernel. The DTI data were acquired using an echo planar imaging sequence with a *b*-value of 1000 s/mm^2^, TR/TE = 8400/64.0 ms, slice thickness of 2.0 mm, voxel size of 2.0 × 2.0 × 2.0 mm^3^, and a total of 75 slices. Subsequently, MRtrix3 [51] was employed for a comprehensive preprocessing procedure, which included denoising, and correction for Gibbs ringing and Rician bias, as well as distortion and eddy current correction. The T1-weighted images, post recon-all processing from FreeSurfer (http://surfer.nmr.mgh.harvard.edu [52]), and subsequent brain extraction were then co-registered with the DTI data. Both the DTI and T1 images were registered to the MNI standard space. Anatomically constrained probabilistic whole-brain fiber tracking was performed, generating a total of 100 million streamlines per subject. This meticulous approach ensured a reliable delineation of white matter tracts throughout the brain.

### Construction of brain networks

The Brainnetome Atlas, which contains 246 subregions of the bilateral hemispheres, was selected for this study due to its multimodal characterization of the human brain. The atlas provides fine-grained subregions based on structural and functional connectivity (FC) patterns, and maps cognitive processes onto these subregions to understand the functional organization of the brain [53].

To assess the static functional connectivity (FC), we computed the Pearson correlation coefficient between the time series of each pair of nodes, resulting in a symmetric 246 by 246 correlation matrix. For the dynamic functional brain network construction, we employed graph theoretical network analysis (GRETNA, http://www.nitrc.org/projects/gretna/ [54]). The estimation of dynamic FC involved the widely adopted sliding-window method, utilizing a window length set at 50 TRs and a step of one TR. This approach yielded a total of 108 windows for each participant, with each window producing a 246 by 246 symmetric correlation matrix. These matrices comprehensively captured the dynamic alterations in FC over the course of the resting-state scan period. Subsequently, these 108 correlation matrices were employed as inputs for further analyses of temporal variability.

Furthermore, to elucidate the structural connectivity (SC) network, we constructed a 246 by 246 SC matrix. This was achieved by aligning each participant’s MNI parcellation scheme to the diffusion space and subsequently scaling it by the inverse of the two node volumes [55]. This detailed approach ensured a robust representation of the anatomical connections within the individual brain network.

### Temporal variability of regional functional architecture

To add the dynamic FC measure, we further investigated the perspective of temporal variability of functional connectivity in each node [30, 56]. For each participant, the nodal temporal variability *V*_*k*_ of node *k* can be described by.

$${V}_{k}= 1 - \frac{{\Sigma }_{i\ne j}{\uprho }_{{F}_{i,k}{F}_{j,k}}}{\text{n}(\text{n}-1)}$$

Where *n* is the total number of windows, and ${\uprho }_{{F}_{i,k}{F}_{j,k}}$ denotes the Pearson correlation coefficient between the FC profiles of region* k* in the correlation matrices derived from the *i* and the *j* windows (*i, j* = 1, 2, …, *n*; *i ≠ j*; *k* = 1, 2, …, 246).

### Brain-age prediction model

The brain-age prediction model employed in this study integrates a multimodal set of neuroimaging features to estimate an individual’s brain age. Specifically, these features encompass (1) SC, which providing insights into the anatomical pathways [57] (2) FC, which indicating synchronized activity across brain regions [58]; (3) regional temporal variability, which offering information on dynamic processes [56]; and (4) regional densities of GM, WM, and CSF, elucidating the distribution of neural and non-neural elements [59].

To evaluate the predictive performance of our models, we implemented a tenfold cross-validation strategy using the KFold class from the sklearn.model_selection module. This method allows for a robust assessment of the model’s generalizability by partitioning the dataset into training and testing subsets, ensuring that each subject is included in both roles across different folds. A fixed random seed (42) was set to maintain reproducibility. We employed Lasso regression [40], specifically the LassoCV function in the scikit-learn library version 1.3.0 [60], which performs automatic hyperparameter tuning to identify the optimal regularization parameter (alpha). During each fold of cross-validation, we trained the model on the training set and evaluated its performance on the testing set. To identify significant features, we examined the coefficients of the Lasso model. Features with non-zero coefficients were deemed relevant, and their indices were recorded. This process was repeated for each fold, and the unique selected feature indices were compiled. Using the identified significant features, we performed a second round of tenfold cross-validation. The Lasso regression approach was applied, focusing solely on the selected features. To evaluate the predictive accuracy of the model, we computed several performance metrics, specifically the mean absolute error (MAE) and root mean squared error (RMSE). These metrics were obtained by comparing the actual chronological ages with the cross-validated age predictions, aggregated across all tenfold [21, 61]. Our primary objective was to identify the model with the lowest MAE across the folds, thereby selecting it as the brain-age prediction model for predicting chronological ages. Additionally, the individual brain-age gap was calculated as the difference between model predicted age and chronological age. All the steps of model training were conducted in Python.

### Network metrics

Investigating the relationship between brain-age gap and graph theory measures for enhanced insight into a deeper understanding of specific node alterations linked to this brain age differential. Graph theory analysis was conducted systematically in the nodal levels in both functional connectivity network and structural connectivity network. Here, negative functional correlations were disregarded, and a binary and undirected functional/structural network was established by applying a 0.1 sparsity threshold to the respective matrices. At the nodal level, we calculated the nodal betweenness centrality, degree centrality, nodal efficiency, and nodal clustering coefficient [62]. Nodal betweenness centrality, calculated as the fraction of shortest paths that traverse a node, assesses a node’s importance in facilitating communication. Degree centrality is determined by counting a node’s connections. Nodal efficiency, computed as the inverse of the harmonic mean of the shortest path lengths, evaluates information exchange efficacy. Nodal clustering coefficient measures the proportion of a node’s neighbors that are also neighbors of each other.

### Statistic analysis

We calculated the correlation between predicted brain age and chronological age, as well as between the brain age gap and chronological age, using the general linear model. Then, we conducted partial correlation analyses to explore the intricate interplay between the brain-age gap, network metrics, and cognitive and emotional assessments. To mitigate potential confounding effects, we included chronological age, gender, and education as nuisance covariates. A false discovery rate (FDR) correction was applied at a significance level of 0.05.

Additionally, the Canonical Correlation Analysis (CCA) was employed to examine the intricate interplay between two distinct sets of variables, facilitating a rigorous assessment of their underlying relationships and potential associations. Firstly, we explored the interrelations between the neurocognitive age-related characteristics and widely used cognitive performance measures (MOCA and AD8) for clinical screening of dementia-related disorders. Then, CCA analyses were conducted to evaluate the interrelations between neurocognitive age-related characteristics and performance-related traits (IADL, PSQI, and LSNS), as well as between neurocognitive age-related characteristics and emotional characteristics (GDS, UCLA_LS, and PSS). The CCA methodology entailed weighing the outcome variables (e.g., neurocognitive age-related canonical variate) most associated with a meticulously weighted combination of the predictor set (e.g., cognitive performance canonical variate). These computations were conducted through linear transformations based on the maximum correlation method [63]. For robust statistical inference, we performed 10,000 permutations of participant indices for the canonical variate scores, generating a null distribution for the correlation value between canonical variates.

In the study cohort, the MOCA scores exhibited a range from 14 to 28. To facilitate a comprehensive analysis, we stratified the participants into two distinct groups based on MOCA scores: one comprising individuals with scores below 18, and the other encompassing those with scores of 25 or higher. This segmentation allowed for a detailed comparison between the group exhibiting the highest MOCA scores (MOCA +) and the one with the lowest (MOCA −). Subsequently, independent-sample *t-*test was employed to explore any significant differences between groups in terms of neuropsychological assessments, and brain-age gap. Furthermore, we conducted k-means clustering with 1000 iterations and a predetermined number of clusters (*k* = 2) to assess the potential to distinguish the MOCA + and MOCA − groups based on an optimal combination of neuropsychological assessments (excluding MOCA), predicted brain age, and brain-age gap. The final accuracy was determined by identifying the most frequently recurring classification across the iterations.

### Validation analysis

We utilized a separate dataset from the Alzheimer’s Disease Neuroimaging Initiative (ADNI, https://adni.loni.usc.edu) [64] cohort to evaluate the performance of our brain-age prediction model. All MRI/DTI data were acquired using a 3-T scanner under consistent settings (i.e., TR = 3000 ms, TE = 30 ms; for details, see https://adni.loni.usc.edu/data-samples/adni-data/neuroimaging/mri/). We extracted all features from this separate dataset using the same analytical methods as in our main study, which included a sample size of 216 with a mean age of 71.31 years (SD = 8.20) within the ADNI cohort. Then, we applied our brain-age prediction model to the separate dataset to evaluate its performance.

Additionally, we employed a dynamic approach emphasizing functional connectivity rather than temporal variability as features for the brain-age prediction model. We utilized the Synchronization Likelihood (SL) method [65] to estimate the functional connectivity between all pairs of brain regions. SL is a dynamic measure of generalized synchronization that assesses the probability of synchronization between two processes at each time point, producing a single 246 by 246 matrix for each subject. Here, we set the following parameters: *m* = 25, *L* = 20, ${w}_{1}$=960, and ${w}_{2}$=1959, while keeping the preferred significance level (${p}_{ref}$) at 0.05, consistent with previous studies [65, 66]. The SL was used as dynamic features instead of temporal variability in training the brain-age prediction model, aiming to determine whether it could influence the model’s construction.

## Results

### Brain-age prediction model 

**Fig. 1:**
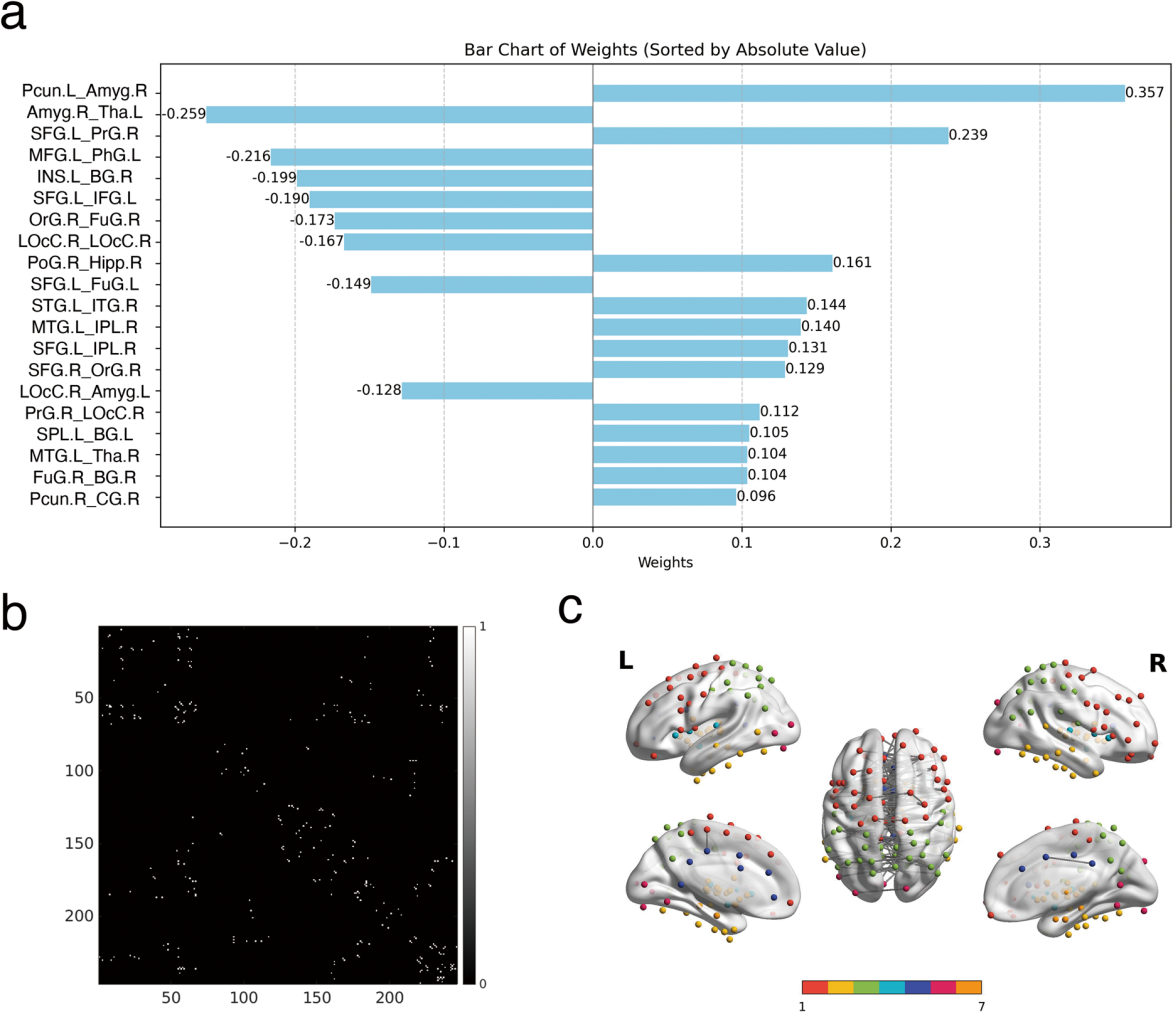
The selected features of the Lasso prediction model. **a** The top 20 features with the highest weights in the brain age prediction model. **b** A 246 × 246 matrix showing structural connections between brain regions, with white indicating connections selected by the brain-age prediction model and black indicating their absence. **c** These structural connectivity features are visually organized into seven distinct brain regions [53]: the frontal lobe (shown in red), temporal lobe (yellow), parietal lobe (green), insular lobe (blue), limbic lobe (purple), occipital lobe (pink), and subcortical nuclei (orange). The gray connections represent the structural connectivity between these brain areas. Several abbreviations to refer to specific brain regions: Pcun (Precuneus), Amyg (Amygdala), Tha (Thalamus), SFG (Superior Frontal Gyrus), PrG (Precentral Gyrus), MFG (Middle Frontal Gyrus), PhG (Parahippocampal Gyrus), INS (Insular Gyrus), BG (Basal Ganglia), IFG (Inferior Frontal Gyrus), OrG (Orbital Gyrus), FuG (Fusiform Gyrus), LOcC (Lateral Occipital Cortex), PoG (Postcentral Gyrus), Hipp (Hippocampus), STG (Superior Temporal Gyrus), ITG (Inferior Temporal Gyrus), MTG (Middle Temporal Gyrus), IPL (Inferior Parietal Lobule), SPL (Superior Parietal Lobule), and CG (Cingulate Gyrus). “R” denotes the right hemisphere, while “L” indicates the left hemisphere

The brain-age prediction model demonstrated strong performance, achieving a MAE of 3.350 and a RMSE of 4.384, with a hyperparameter value of alpha set to 0.019. These impressive performance features underscore the model’s accuracy and its potential utility in estimating brain age based on multimodal neuroimaging data, including FC, SC, and measures of brain tissue density and temporal regional variability. In our analysis, we identified the top 20 features with the highest weights (Fig. 1a). The most significant features were predominantly located within the basal ganglia, thalamus, and orbital gyrus. Notably, the strongest connection was observed between the right basal ganglia and the left parahippocampal gyrus, exhibiting a weight of 0.357. This was followed by the connection between the right thalamus and the left parahippocampal gyrus, which had a weight of − 0.239. Additional important features emerged from the thalamic and orbitofrontal regions. The Fig. 1b presents the final selection of all 159 features that significantly contribute to the prediction model, particularly in the context of structural connectivity (SC). The analysis revealed that these connections predominantly involved interactions between the right and left hemispheres, especially within the frontal, temporal, and parietal lobes. This emphasized on inter-hemispheric SC underscored its pivotal contribution to the accuracy of the brain-age prediction model.

**Fig. 2:**
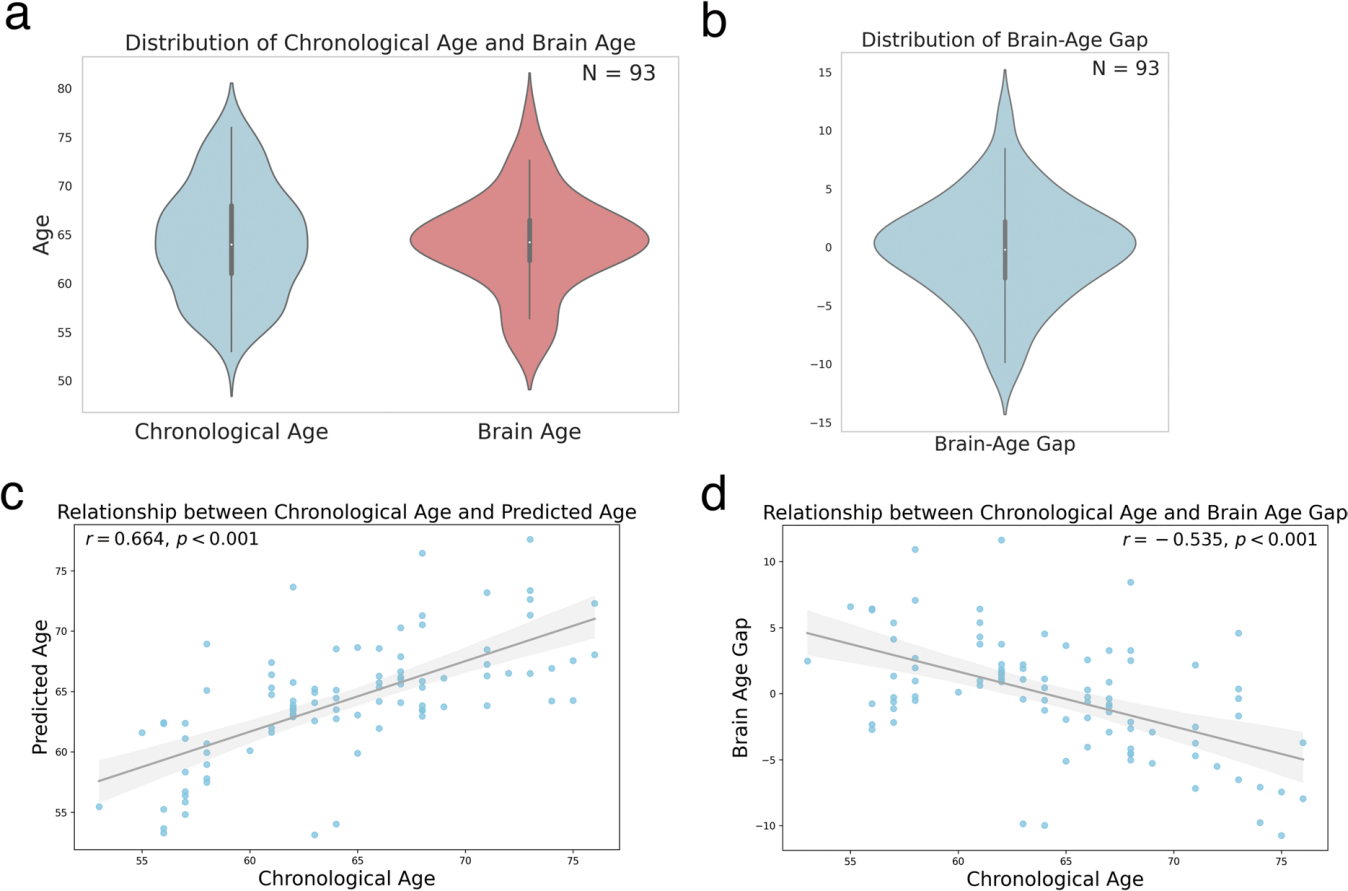
Distribution and correlation analysis of chronological age, brain age and brain-age gap. **a** Violin plot depicting the distribution of chronological age (blue) and brain age (red) among the study participants (*N* = 93). **b** The distribution of the brain-age gap. **c** The positive correlation between chronological age and predicted brain age, with a Pearson correlation coefficient of *r* = 0.664 and *p* < 0.001. **d** The negative correlation between chronological age and the brain-age gap (*r* = − 0.535, *p* < 0.001). Each node represents data from an individual participant. The shaded area in both scatter plots represents the 95% confidence interval for the regression line

We further delineated the distribution of brain age, chronological age, and the brain-age gap, as illustrated in Fig. 2a–b. We conducted a Pearson correlation analysis to assess the relationship between chronological age and brain age (*r* = 0.664, *p* < 0.001, Fig. 2c). Similarly, we examined the correlation between chronological age and the brain-age gap (*r* = − 0.535, *p* < 0.001, Fig. 2d). These analyses provide insights into the associations among these variables, contributing to our understanding of the aging process in the brain.

### The relationship between brain-age gap and network metrics

**Fig. 3:**
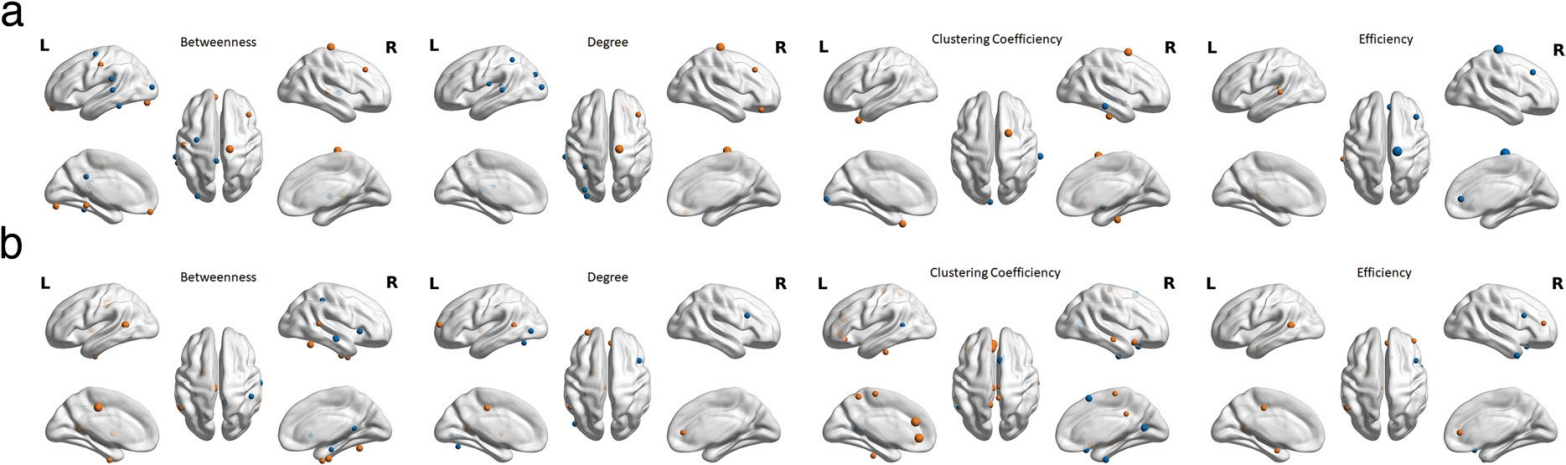
The noteworthy correlations between brain-age gap and **a** the nodal characteristics within the functional network, as well as **b** the nodal characteristics within the structural network. In this representation, negative correlations are indicated by nodes in blue, while positive correlations are represented by nodes in orange. The size of each node indicated the *rho* value. For detailed nodal information, please refer to Tables S1 and S2

Previous research has demonstrated that functional connectivity may undergo changes while structural connectivity remains relatively stable during tasks [67, 68]. Although structural connectivity had the most contribution to predicted brain age, the functional connectivity may also provide additional information compared to structural connectivity. For this reason, the partial correlation analyses conducted in this study revealed notable associations between the brain-age gap and network metrics at various nodal levels within both the functional and structural connectivity networks. In terms of functional network metrics, we observed a diverse range of both negative and positive correlations in nodal characteristics, including nodal betweenness centrality, degree centrality, nodal clustering coefficient, and nodal efficiency (Fig. 3a, Table S1). Negative correlations were particularly pronounced in regions such as the basal ganglia, superior/middle temporal gyrus, inferior/superior parietal lobule, lateral occipital cortex, insular gyrus, and cingulate gyrus (*rho* = [− 0.209, − 0.257], *p* < 0.05, non-FDR corrected). Conversely, positive correlations were noted in areas like the parahippocampal gyrus, orbital gyrus, and posterior middle/superior frontal gyrus (*rho* = [0.210, 0.334], *p* < 0.05, non-FDR corrected). Notably, the nodal efficiency of the precentral gyrus exhibited a significant positive correlation with the brain-age gap (*rho* = 0.398, *p* < 0.05, FDR corrected).

In the context of the structural network, we observed similar patterns of both negative and positive correlations across all nodal characteristics (Fig. 3b, Table S2). Negative associations were identified in regions including the superior frontal gyrus, middle temporal gyrus, inferior parietal lobule, parahippocampal gyrus, and lateral occipital cortex (*rho* = [− 0.209, − 0.294], *p* < 0.05, non-FDR corrected). Conversely, positive correlations were noted in the cingulate gyrus, basal ganglia, posterior superior temporal sulcus, and middle frontal gyrus (*rho* = [0.210, 0.363], *p* < 0.05, non-FDR corrected). Furthermore, the cingulate gyrus exhibited significant positive correlations in all nodal characteristics with the brain-age gap. Taken together, these results emphasize a unique interaction between the mentioned regions and the aging process.

### The relationship between brain-age gap and resilience

**Fig. 4:**
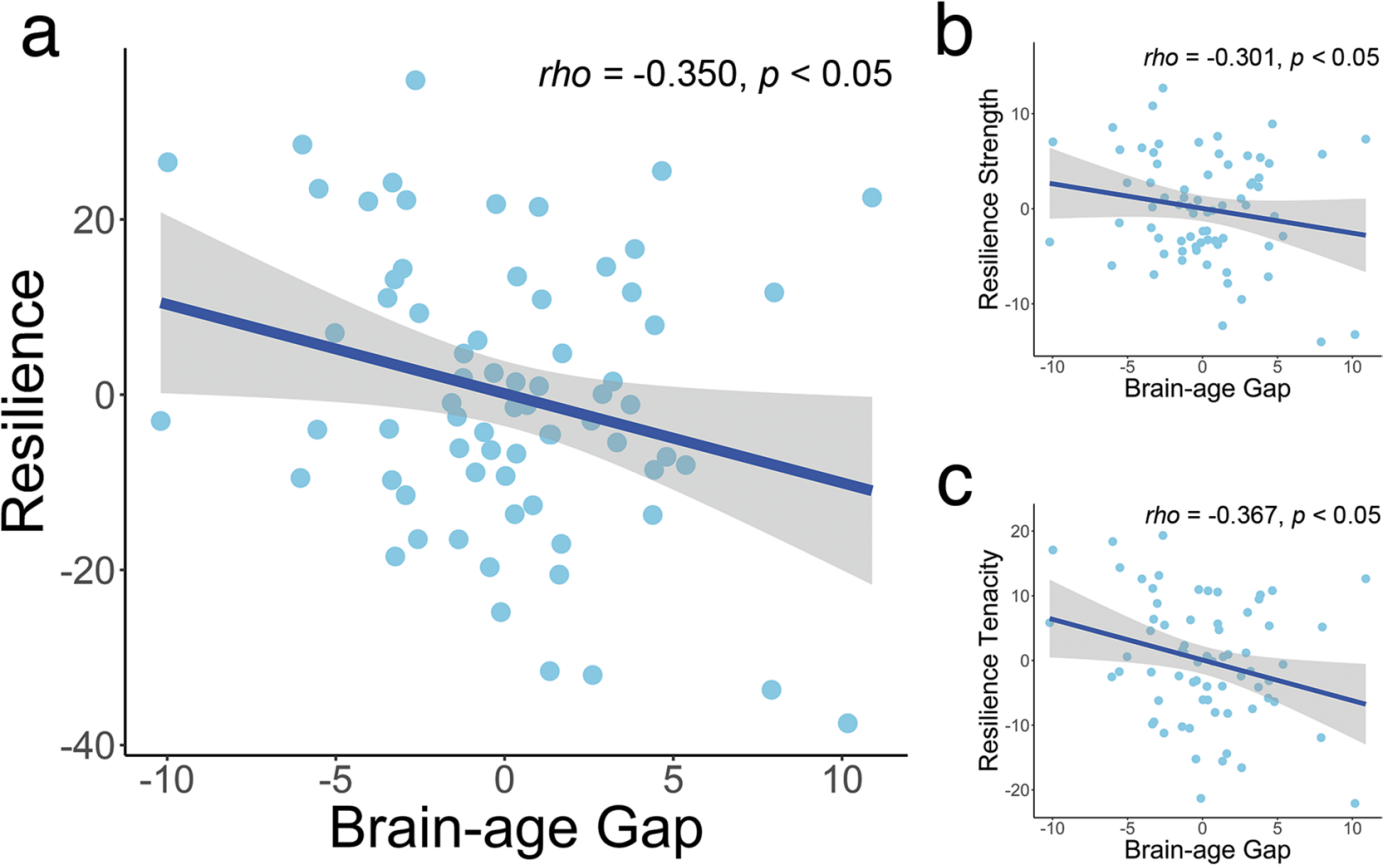
The partial correlations between brain-age gap and resilience measures. The partial correlation between the brain-age gap and **a** overall resilience; **b** tenacity of resilience; **c** strength of resilience, controlling for age, gender, and education. The data points represent individual subject data. The blue lines depict the partial correlation fits, and the gray shaded areas surrounding the lines indicate the standard deviation

Using the partial correlation, our analysis revealed a significant negative correlation between psychological resilience and brain-age gap (*rho* = − 0.350, *p* < 0.05, Fig. 4a). Specifically, both the tenacity (*rho* = − 0.367, *p* < 0.05) and strength components of resilience (*rho* = − 0.301, *p* < 0.05, Fig. 4b) exhibited negative correlations with brain-age gap. These findings suggest that higher levels of resilience are associated with a reduced disparity between predicted brain age and chronological age.

### Canonical variates associated with brain-age gap in CCA analysis

**Fig. 5:**
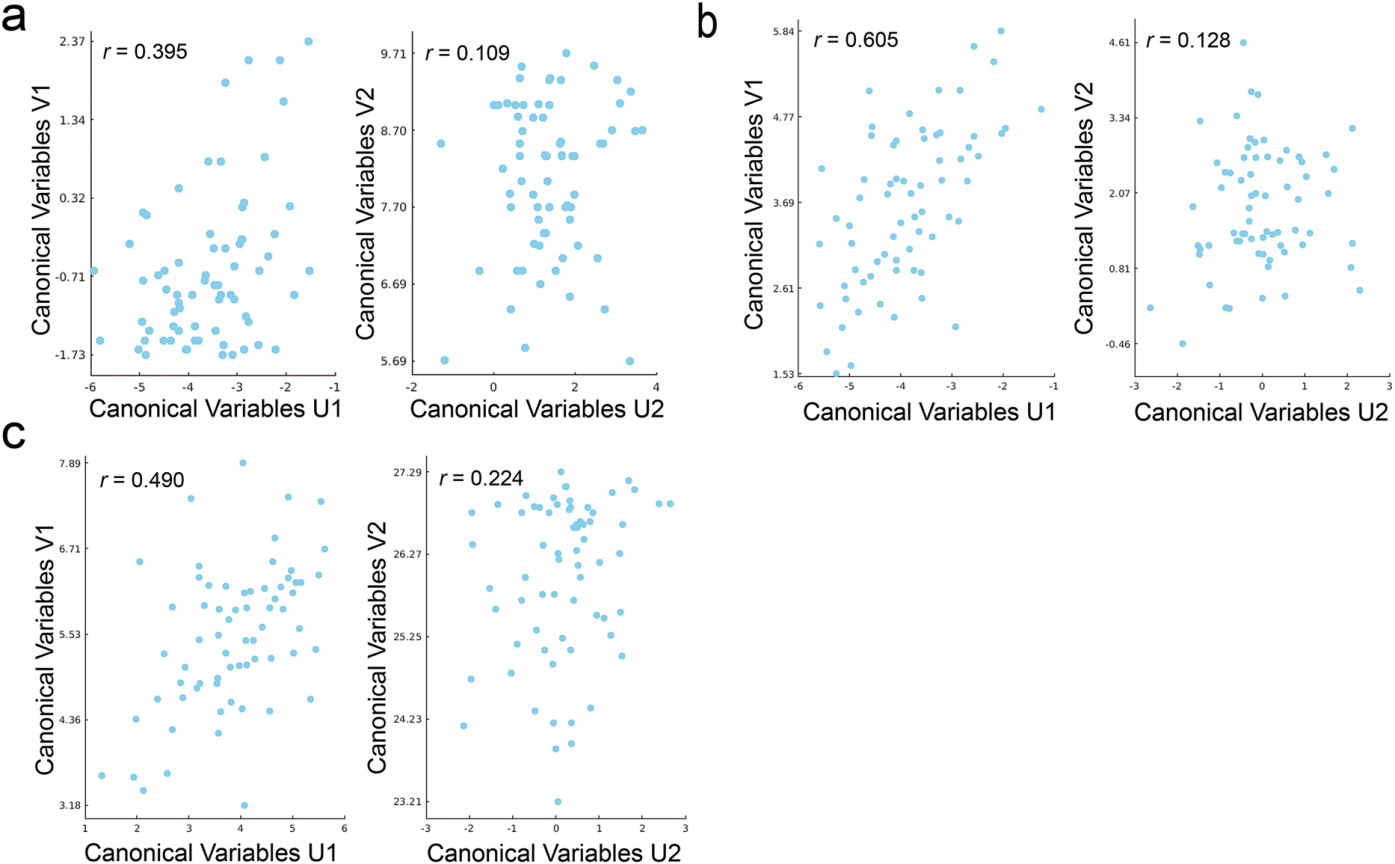
Canonical Correspondence Analysis (CCA) results comparing different variable pairs. **a** CCA between neurocognitive age-related canonical variate and cognitive performance canonical variate, showing canonical variables U1 vs. V1 (*r* = 0.305, *p* = 0.109) higher than U2 vs V2. **b** CCA between neurocognitive age-related variate and performance-related variate with canonical variables U1 vs. V1 (*r* = 0.606, *p* < 0.001) higher than U2 vs V2. **c** CCA between neurocognitive age-related variate and emotional variate, displaying U1 vs. V1 (*r* = 0.840, *p* < 0.001) higher than U2 vs V2. Each node represents data from an individual participant

By applying CCA, we only considered the first pair of canonical variates which established the maximum association between two canonical variates [69]. The association between the first pair of neurocognitive age-related canonical variate and cognitive performance canonical variate (*r* = 0.395, *p* < 0.05, 10,000 permutations, Fig. 5a), which showed a mutual influence between these two variables. A cutoff of 0.3 was applied to the canonical structure coefficient to differentiate between large and small contributions [70]. In this context, the structure coefficients (*r*_*s*_) of brain-age gap and resilience were − 0.365 and − 0.859, respectively, underscoring their significant contributions to the neurocognitive age-related canonical variate. Besides, MOCA and AD8 exhibited structure coefficients of − 0.684 and 0.983, respectively, further emphasizing their relevance as criteria for the neurocognitive age-related canonical variate. In our subsequent CCA analyses, we found compelling correlations between the first pair of neurocognitive age-related variate and both the performance-related variate (*r* = 0.490, *p* < 0.05, 10,000 permutations, Fig. 5b) and the emotional variate (*r* = 0.605, *p* < 0.001, 10,000 permutations, Fig. 5c). Upon indictors of neurocognitive age-related variate, we found that brain-age gap had no significant contributions to both performance-related (*r*_*s*_ = 0.029) and emotional variate (*r*_*s*_ = 0.054). In contrast, Resilience emerged as a key factor, demonstrating substantial contributions to both performance-related (*r*_*s*_ = − 0.997) and emotional variate (*r*_*s*_ = 0.988), where GDS (*r*_*s*_ = 0.642), UCLA_LS (*r*_*s*_ = 0.672), PSS (*r*_*s*_ = 0.821), PSQI (*r*_*s*_ = − 0.066), IADL (*r*_*s*_ = 0.177), and LSNS (*r*_*s*_ = 0.995), respectively.

### Brain-age gap based classification of MOCA levels

Using independent-sample *t*-tests, we observed significant differences across various assessments. Specifically, the MOCA scores demonstrated a substantial distinction (*t* = − 19.622, *p* < 0.001), indicating notably higher scores in the MOCA + group compared to the MOCA − group. Similarly, the AD8 scores exhibited a substantial variation (*t* = 5.300, *p* < 0.001). Additionally, significant between-group differences were observed in Resilience scores (*t* = − 2.413, *p* < 0.05), as well as its subcomponents: strength of Resilience (*t* = − 2.643, *p* < 0.05) and optimism of Resilience (*t* = − 2.393, *p* < 0.05). These findings collectively affirm that the MOCA + group demonstrated notably better performance compared to the MOCA − group across various cognitive and resilience measures.

**Fig. 6:**
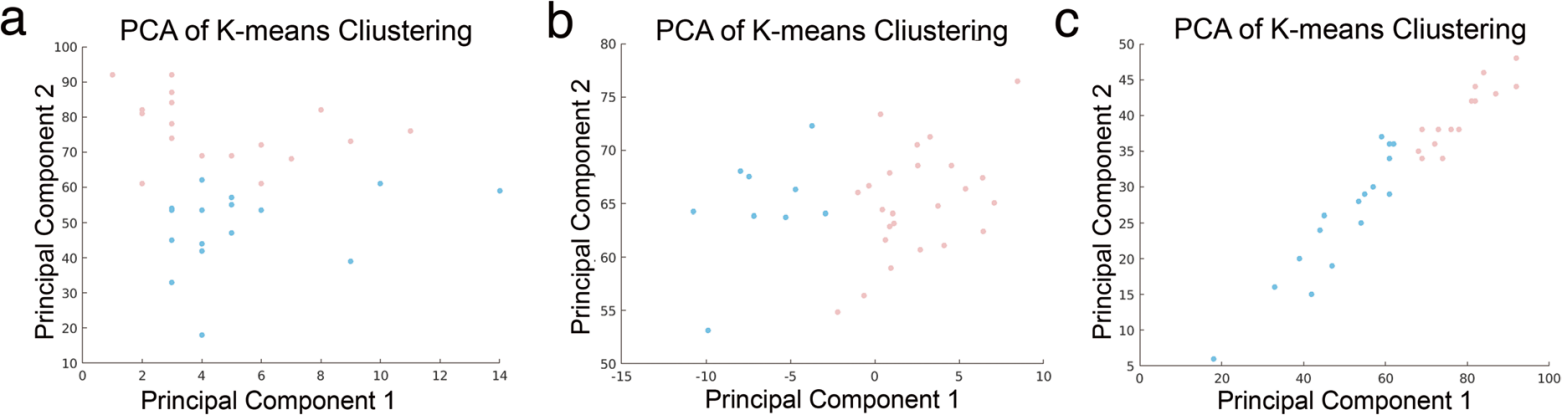
The *k*-means clustering results visualized with principal component analysis. **a** Classification accuracy of cognitive and emotional performance scores (excluding MOCA) alongside the brain-age gap, achieving an accuracy of 0.788. **b** Classification accuracy using both brain-age gap and Resilience features, maintaining a high accuracy of 0.758. **c** Classification accuracy with only the Resilience feature, resulting in an accuracy of 0.727. Each node represents a participant, and are colored to distinguish between clusters

Additionally, the k-means clustering was used to partition subjects into two distinct groups based on different combinations of features. The classification accuracies varied depending on the included features. When considering all cognitive and emotional performance scores (without MOCA), and brain-age gap, the classification accuracy was 0.788 (Fig. 6a). Using both brain-age gap and Resilience or only brain-age gap as the feature, the classification accuracies are equally remained high at 0.758 (Fig. 6b). Using only Resilience as the feature, the classification accuracy was 0.727 (Fig. 6c).

### Validation results

In our evaluation of the brain-age prediction model using a separate dataset from the Alzheimer’s Disease Neuroimaging Initiative (ADNI) cohort, we observed a MAE of 8.876 and a RMSE of 11.258, which were not as favorable as the results obtained in our primary study. While our brain-age prediction model utilizing dynamic features derived from SL achieved a MAE of 4.244 and a RMSE of 5.319, with the hyperparameter alpha optimized at 3.875, its performance was not as favorable as that reported in the main text (refer to the Supplementary for more details).

## Discussion

The brain-age gap is a widely used index indicating a potential difference between an individual’s predicted brain age and chronological age, offering insights into the senescence process [71]. Higher than chronological age is thought partially to reflect an above-average rate of ageing. Utilizing the Lasso regression model, this study identified significant features exclusively from structural connectivity data, providing initial evidence to suggest the potential of the brain-age gap as a promising ageing marker. It highlights the role of resilience as a protective factor against the ageing process. The brain-age prediction model’s most important features were based on structural connectivity. After calculating the brain-age gap, it showed associations with various nodal characteristics, such as nodal betweenness centrality, in both the functional and structural connectivity network. Furthermore, a negative correlation was observed between the brain-age gap and resilience. Through the application of the CCA approach, our findings demonstrated a significant association between the neurocognitive age-related canonical variate (brain-age gap and resilience) and the cognitive performance canonical variate (MOCA and AD8), indicating a mutual influence between these two canonical variates. Moreover, the MOCA + group demonstrated notably better resilience than the MOCA − group. Both the brain-age gap and resilience demonstrated the ability to classify individuals into MOCA + and MOCA − groups with an accuracy exceeding 0.72. In summary, this research reveals the significant roles of the brain-age gap and resilience in understanding cognitive health among older individuals.

The brain-age prediction model highlights the significant role played by structural connectivity features, especially those facilitating interactions between the right and left hemispheres. Previous research has demonstrated that inter-hemispheric structural connectivity is positively related to syntactic performance in older and younger adults, emphasizing its importance for syntactic functions [72]. Moreover, the decline in inter-hemispheric structural connectivity initiates around the age of approximately 48 years old [73], attributed to the physiological age-related reduction in the size and micro-structural integrity of the corpus callosum [74, 75]. Additionally, the identified structural features were primarily associated with the sensorimotor network and default mode network. This observation aligns with prior studies indicating age-related decreases in inter-hemispheric integration within these specific networks [76, 77]. It also implies that inter-hemispheric structural connectivity could function as a sensitive biomarker for aging, potentially reflecting changes in language ability during the aging process. This underscores the significance of delving deeper into the complexities of inter-hemispheric structural connectivity to gain a better understanding of age-related cognitive processes. Additionally, the most influential features in brain age prediction are the structural connections between the left precuneus and the right amygdala, as well as between the right amygdala and the left thalamus. These connections highlight the significant role of the amygdala in brain age, suggesting that alterations in these pathways may reflect broader changes in emotional and cognitive processing as individuals age [78, 79].

The associations between the brain-age gap and nodal characteristics were identified within both the functional and structural connectivity networks. Nodal characteristics, including betweenness centrality, degree, clustering coefficient, and efficiency, collectively underscore the pivotal role of a node in efficiently connecting and disseminating information throughout the network, highlighting its overall significance [62, 80, 81]. In our findings, regions consistently exhibited significant negative correlations in functional and structural nodal characteristics with the brain-age gap, including the middle temporal gyrus, inferior/superior parietal lobule, and lateral occipital cortex. This consistency may be attributed to the close interconnection between these three regions in functional and structural connectivity [82, 83]. Additionally, previous studies suggest that the temporal gyrus, parietal lobule, and occipital cortex are all associated with attention processes [82–85]. Indeed, the observed negative association between attention processes and the brain-age gap suggests that as the predicted brain age surpasses the chronological age, there may be a decline in individual attention performance. This finding underscores the potential impact of ageing-related changes on cognitive functions, particularly in tasks requiring focused attention [86]. Furthermore, we propose that the brain-age gap could be a significant feature for assessing the attention changes associated with ageing.

The region consistently positively correlated with the brain-age gap is the middle frontal gyrus, which plays a role in attention, working memory, and language-related processing [87]. Previous suggestions have proposed that the middle frontal gyrus may act as a compensatory mechanism for reduced medial temporal activity, which could be a result of declining attentional capabilities and cognitive deficits associated with aging [88, 89]. Additionally, the frontal gyrus exhibits increased redundancy, indicating changes in the higher-order informational organization of the aging brain [13]. Interestingly, the parahippocampal gyrus demonstrated significant negative correlations with a brain-age gap in the functional network but displayed positive correlations in the structural network. The cingulate gyrus exhibited significant negative correlations in all nodal characteristics with the brain-age gap in the structural network while yielding positive results in the functional network. The observed inconsistency in results between the functional and structural networks may be attributed to the inherent differences in the structures of these networks, a notion supported by previous studies indicating a rapid reconfiguration of whole-brain functional connectivity while structural connectivity remains relatively stable [67, 68]. Compensatory mechanisms could potentially explain these positive correlations in both regions. From a biological ageing standpoint, the onset of age-related functional decline and subsequent frailty is likely to initiate when compensatory mechanisms are depleted, diminishing the organism’s resilience [90]. In-depth investigation of the intricate roles of the parahippocampal and cingulate gyri during ageing will offer insight into the underpinnings of neurodegenerative changes. Additionally, both regions are parts of the default mode network that correlates with individual cognitive performances (e.g., working memory, attention) [91–93]. Our findings suggest that the brain-age gap, potentially a composite feature, requires further exploration for its sensitivity to specific cognitive functions such as working memory.

There was a significant negative correlation between resilience and the brain-age gap, suggesting that individuals with higher levels of resilience tend to exhibit a reduced disparity between their predicted brain age and chronological age. Notably, a more significant brain-age gap is associated with higher mortality [4], and our findings hint at a potential link between this association and lower resilience. In line with this, our results add evidence to confirm the potential benefits of promoting resilience in older adults [37, 38]. Moreover, previous studies have identified a potential association between a higher brain-age gap and lower life satisfaction in ageing, which may be related to resilience [35]. Our findings found that some significant features related to brain-age gap. Future research could investigate the underlying mechanisms of these relationships to better understand how specific brain features influence resilience, potentially guiding interventions aimed at preventing cognitive decline.

In our study, Canonical Correlation Analysis revealed a strong link between neurocognitive age-related and cognitive performance, suggesting their potential as valuable predictors. As indicated by canonical structure coefficients, the influential contributions of all factors in each variate suggest a significant association between the brain-age gap and cognitive function. Considering the influence of various lifestyle factors, the brain-age gap holds promise as a potential marker for age-related cognitive decline [9, 94]. Moreover, our findings highlight the important role of resilience in both cognitive and emotional performances. The MOCA + group demonstrated better resilience than the MOCA − group, underscoring the potential importance of enhancing resilience for promoting cognitive health [95]. Although incorporating features such as the brain-age gap and cognitive and emotional performances enhance accuracy beyond using solely the brain-age gap and resilience, it requires more time to obtain these varied features. Either the brain-age gap or resilience demonstrated strong classification abilities, accurately distinguishing individuals into MOCA + and MOCA − groups with over 72% accuracy. This indicates their potential as screening index for assessing cognitive functioning. The generalizability of using these two indicators in future applications remains to be discussed. Resilience is commonly evaluated through questionnaire. The reliance on subject self-reports on resilience questionnaire renders it higher reliability threat than objective measurement of the brain age gap. Including other cognitive and affective assessments in the prediction modelling show a trivial improvement in the classification accuracy and is considered not cost-effective taking into the consideration of time and human resources invested in conducting these assessments.

The present work has limitations that should be noted. While our findings suggest the potential utility of the brain-age gap as an ageing biomarker and highlight the protective role of resilience, it is essential to acknowledge that further validation in larger, diverse datasets and other existing models is warranted to establish the robustness and generalizability of these associations. This additional validation will further our understanding of the potential of the brain-age gap as a reliable biomarker for assessing aging-related changes in neurocognitive functioning. In this study, it is acknowledged that MOCA and AD8 were not used to categorize individuals into healthy control or mild cognitive impairment groups due to their inconsistent group divisions. Previous studies have also indicated that these measures may have different sensitivity to group division, but none are infallible [96]. The brain-age gap and resilience effectively distinguished between MOCA + and MOCA − groups in this study, emphasizing their importance in ageing research. Future studies could explore integrating other cognitive assessments to fine-tuned group categorization and enhance cognitive evaluation precision in ageing research. Additionally, our study revealed a negative relationship between resilience and the brain-age gap, indicating that resilience is a protective factor against the ageing process. Previous research has shown that resilience can be enhanced [39] and may help close the brain-age gap. This presents an intriguing avenue for future research to explore in greater detail, providing valuable insights into interventions to promote cognitive health during the ageing process.

## Conclusion

The brain-age gap as a potential index reflects the status of brain health. Together with psychological resilience, which appears to protect against premature ageing of the brain, they classified older people’s neurocognitive status measured by the MOCA scores with high accuracy. These findings suggest that brain-age gap is useful for screening for premature ageing, allowing for timely interventions including programmes to build psychological resilience.

## Supplementary Information

Below is the link to the electronic supplementary material.

Supplementary file1 (DOCX 2803 KB)

## Data Availability

The processed data and code utilized in this study can be made available upon a reasonable request. For any requests or needs regarding the data and code, we encourage early communication with the corresponding author to obtain the necessary permissions for collaboration. The raw data are not publicly available due to a lack of informed consent from the participants and ethical approval for public data sharing.

## References

[1] López-Otín C, Blasco MA, Partridge L, Serrano M, Kroemer G. The hallmarks of aging. Cell. 2013;153:1194–217. 10.1016/j.cell.2013.05.039. 23746838 10.1016/j.cell.2013.05.039 PMC3836174

[2] Ludwig FC, Smoke ME. The measurement of biological age. Exp Aging Res. 1980;6:497–522. 10.1080/03610738008258384. 7011818 10.1080/03610738008258384

[3] Franke K, Ziegler G, Klöppel S, Gaser C. Estimating the age of healthy subjects from T1-weighted MRI scans using kernel methods: exploring the influence of various parameters. Neuroimage. 2010;50:883–92. 10.1016/j.neuroimage.2010.01.005. 20070949 10.1016/j.neuroimage.2010.01.005

[4] Cole JH, Ritchie SJ, Bastin ME, Valdés Hernández MC, Muñoz Maniega S, Royle N, et al. Brain age predicts mortality. Mol Psychiatry. 2018;23:1385–92. 10.1038/mp.2017.62. 28439103 10.1038/mp.2017.62 PMC5984097

[5] Franke K, Klöppel S, Koutsouleris N, Davatzikos C, Sauer H, Gaser C. BrainAGE scores derived from structural MRI predict conversion from MCI to AD. RöFo - Fortschritte Auf Dem Geb Röntgenstrahlen Bildgeb Verfahr. 2010;182:A6. 10.1055/s-0030-1268271.

[6] Gaser C, Franke K, Klöppel S, Koutsouleris N, Sauer H, Initiative ADN. BrainAGE in mild cognitive impaired patients: predicting the conversion to Alzheimer’s disease. PLoS ONE. 2013;8:e67346. 10.1371/journal.pone.0067346. 23826273 10.1371/journal.pone.0067346 PMC3695013

[7] Gonneaud J, Baria AT, Pichet Binette A, Gordon BA, Chhatwal JP, Cruchaga C, et al. Accelerated functional brain aging in pre-clinical familial Alzheimer’s disease. Nat Commun. 2021;12:5346. 10.1038/s41467-021-25492-9. 34504080 10.1038/s41467-021-25492-9 PMC8429427

[8] Baker GT, Sprott RL. Biomarkers of aging. Exp Gerontol. 1988;23:223–39. 10.1016/0531-5565(88)90025-3. 3058488 10.1016/0531-5565(88)90025-3

[9] Cole JH, Marioni RE, Harris SE, Deary IJ. Brain age and other bodily ‘ages’: implications for neuropsychiatry. Mol Psychiatry. 2019;24:266–81. 10.1038/s41380-018-0098-1. 29892055 10.1038/s41380-018-0098-1 PMC6344374

[10] Burke SN, Barnes CA. Neural plasticity in the ageing brain. Nat Rev Neurosci. 2006;7:30–40. 10.1038/nrn1809. 16371948 10.1038/nrn1809

[11] Grady C. The cognitive neuroscience of ageing. Nat Rev Neurosci. 2012;13:491–505. 10.1038/nrn3256. 22714020 10.1038/nrn3256 PMC3800175

[12] Gu Y, Li L, Zhang Y, Ma J, Yang C, Xiao Y, et al. The overlapping modular organization of human brain functional networks across the adult lifespan. Neuroimage. 2022;253:119125. 10.1016/j.neuroimage.2022.119125. 35331872 10.1016/j.neuroimage.2022.119125

[13] Gatica M, Cofre R, Mediano PAM, Rosas FE, Patricio O, Diez I, et al. High-order interdependencies in the aging brain. Brain Connect. 2020;11:734–44. 10.1089/brain.2020.0982. 10.1089/brain.2020.0982 33858199

[14] Bassett DS, Sporns O. Network neuroscience. Nat Neurosci. 2017;20:353. 10.1038/nn.4502. 28230844 10.1038/nn.4502 PMC5485642

[15] Cao M, Wang JH, Dai ZJ, Cao XY, Jiang LL, Fan FM, et al. Topological organization of the human brain functional connectome across the lifespan. Dev Cogn Neurosci. 2014;7:76–93. 10.1016/j.dcn.2013.11.004. 24333927 10.1016/j.dcn.2013.11.004 PMC6987957

[16] Dubois J, Adolphs R. Building a science of individual differences from fMRI. Trends Cogn Sci. 2016;20:425–43. 10.1016/j.tics.2016.03.014. 27138646 10.1016/j.tics.2016.03.014 PMC4886721

[17] Vaher K, Galdi P, Blesa Cabez M, Sullivan G, Stoye DQ, Quigley AJ, et al. General factors of white matter microstructure from DTI and NODDI in the developing brain. Neuroimage. 2022;254:119169. 10.1016/j.neuroimage.2022.119169. 35367650 10.1016/j.neuroimage.2022.119169

[18] Anatürk M, Kaufmann T, Cole JH, Suri S, Griffanti L, Zsoldos E, et al. Prediction of brain age and cognitive age: quantifying brain and cognitive maintenance in aging. Hum Brain Mapp. 2021;42:1626–40. 10.1002/hbm.25316. 33314530 10.1002/hbm.25316 PMC7978127

[19] Bashyam VM, Erus G, Doshi J, Habes M, Nasralah I, Truelove-Hill M, et al. MRI signatures of brain age and disease over the lifespan based on a deep brain network and 14 468 individuals worldwide. Brain. 2020;143:2312–24. 10.1093/brain/awaa160. 32591831 10.1093/brain/awaa160 PMC7364766

[20] Cole JH, Franke K. Predicting age using neuroimaging: innovative brain ageing biomarkers. Trends Neurosci. 2017;40:681–90. 10.1016/j.tins.2017.10.001. 29074032 10.1016/j.tins.2017.10.001

[21] Lund MJ, Alnæs D, de Lange A-MG, Andreassen OA, Westlye LT, Kaufmann T. Brain age prediction using fMRI network coupling in youths and associations with psychiatric symptoms. NeuroImage Clin. 2022;33:102921. 10.1016/j.nicl.2021.102921. 34959052 10.1016/j.nicl.2021.102921 PMC8718718

[22] Han H, Ge S, Wang H. Prediction of brain age based on the community structure of functional networks. Biomed Signal Process Control. 2023;79:104151. 10.1016/j.bspc.2022.104151.

[23] Li H, Satterthwaite TD, Fan Y (2018) Brain age prediction based on resting-state functional connectivity patterns using convolutional neural networks. 2018 IEEE 15th Int. Symp. Biomed. Imaging ISBI 2018, Washington, DC: IEEE. p. 101–4. 10.1109/ISBI.2018.8363532. 10.1109/ISBI.2018.8363532 PMC6074039 30079125

[24] Mwangi B, Hasan KM, Soares JC. Prediction of individual subject’s age across the human lifespan using diffusion tensor imaging: a machine learning approach. Neuroimage. 2013;75:58–67. 10.1016/j.neuroimage.2013.02.055. 23501046 10.1016/j.neuroimage.2013.02.055

[25] Wang L, Su L, Shen H, Hu D. Decoding lifespan changes of the human brain using resting-state functional connectivity MRI. PLoS ONE 2012;7. 10.1371/journal.pone.0044530 10.1371/journal.pone.0044530 PMC3431403 22952990

[26] Zhai J, Li K. Predicting brain age based on spatial and temporal features of human brain functional networks. Front Hum Neurosci. 2019;13:62. 10.3389/fnhum.2019.00062. 30863296 10.3389/fnhum.2019.00062 PMC6399206

[27] Bonifazi P, Erramuzpe A, Diez I, Gabilondo I, Boisgontier MP, Pauwels L, et al. Structure–function multi-scale connectomics reveals a major role of the fronto-striato-thalamic circuit in brain aging. Hum Brain Mapp. 2018;39:4663–77. 10.1002/hbm.24312. 30004604 10.1002/hbm.24312 PMC6866396

[28] Allen EA, Damaraju E, Plis SM, Erhardt EB, Eichele T, Calhoun VD. Tracking whole-brain connectivity dynamics in the resting state. Cereb Cortex. 2014;24:663–76. 10.1093/cercor/bhs352. 23146964 10.1093/cercor/bhs352 PMC3920766

[29] Chang C, Glover G. Time–frequency dynamics of resting-state brain connectivity measured with fMRI. Neuroimage. 2010;50:81–98. 10.1016/j.neuroimage.2009.12.011. 20006716 10.1016/j.neuroimage.2009.12.011 PMC2827259

[30] Gu Y, Lin Y, Huang L, Ma J, Zhang J, Xiao Y, et al. Abnormal dynamic functional connectivity in Alzheimer’s disease. CNS Neurosci Ther. 2020;26:962–71. 10.1111/cns.13387. 32378335 10.1111/cns.13387 PMC7415210

[31] Kaiser RH, Whitfield-Gabrieli S, Dillon DG, Goer F, Beltzer M, Minkel J, et al. Dynamic resting-state functional connectivity in major depression. Neuropsychopharmacology. 2016;41:1822–30. 10.1038/npp.2015.352. 26632990 10.1038/npp.2015.352 PMC4869051

[32] Madsen W, Ambrens M, Ohl M. Enhancing resilience in community-dwelling older adults: a rapid review of the evidence and implications for public health practitioners. Front Public Health 2019;7. 10.3389/fpubh.2019.00014 PMC6374312 30792974

[33] Rentz DM, Mormino EC, Papp KV, Betensky RA, Sperling RA, Johnson KA. Cognitive resilience in clinical and preclinical Alzheimer’s disease: the association of amyloid and tau burden on cognitive performance. Brain Imaging Behav 2017;11:383–90. 10/gsgvdp. 10.1007/s11682-016-9640-4 PMC5391311 27738998

[34] Holz NE, Tost H, Meyer-Lindenberg A. Resilience and the brain: a key role for regulatory circuits linked to social stress and support. Mol Psychiatry. 2020;25:379–96. 10.1038/s41380-019-0551-9. 31628419 10.1038/s41380-019-0551-9

[35] Jawinski P, Markett S, Drewelies J, Düzel S, Demuth I, Steinhagen-Thiessen E, et al. Linking brain age gap to mental and physical health in the Berlin Aging Study II. Front Aging Neurosci. 2022;14:791222. 10.3389/fnagi.2022.791222. 35936763 10.3389/fnagi.2022.791222 PMC9355695

[36] Jeste DV, Savla GN, Thompson WK, Vahia IV, Glorioso DK, Martin AS, et al. Association between older age and more successful aging: critical role of resilience and depression. Am J Psychiatry. 2013;170:188–96. 10.1176/appi.ajp.2012.12030386. 23223917 10.1176/appi.ajp.2012.12030386 PMC3593664

[37] Fontes AP, Neri AL. Resilience in aging: literature review. Ciênc Saúde Coletiva. 2015;20:1475–95. 10.1590/1413-81232015205.00502014. 10.1590/1413-81232015205.00502014 26017950

[38] Shen K, Zeng Y. The association between resilience and survival among Chinese elderly. In: Resnick B, Gwyther LP, Roberto KA, editors. Resil. Aging Concepts Res. Outcomes, New York, NY: Springer; 2011, p. 217–29. 10.1007/978-1-4419-0232-0_14.

[39] Griffith J, West C. Master resilience training and its relationship to individual well-being and stress buffering among army national guard soldiers. J Behav Health Serv Res. 2013;40:140–55. 10.1007/s11414-013-9320-8. 23494766 10.1007/s11414-013-9320-8

[40] Tibshirani R. Regression shrinkage and selection via the Lasso. J R Stat Soc Ser B Methodol. 1996;58:267–88. 10.1111/j.2517-6161.1996.tb02080.x.

[41] Yu X, Zhang J. Factor analysis and psychometric evaluation of the Connor-Davidson Resilience Scale (CD-RISC) with Chinese People. Soc Behav Personal Int J. 2007;35:19–30. 10.2224/sbp.2007.35.1.19.

[42] Fang Y, Tao Q, Zhou X, Chen S, Huang J, Jiang Y, et al. Patient and family member factors influencing outcomes of poststroke inpatient rehabilitation. Arch Phys Med Rehabil. 2017;98:249-255.e2. 10.1016/j.apmr.2016.07.005. 27475119 10.1016/j.apmr.2016.07.005

[43] Buysse DJ, Reynolds CF, Monk TH, Berman SR, Kupfer DJ. The Pittsburgh sleep quality index: a new instrument for psychiatric practice and research. Psychiatry Res. 1989;28:193–213. 10.1016/0165-1781(89)90047-4. 2748771 10.1016/0165-1781(89)90047-4

[44] Lawton MP, Brody EM. Assessment of older people: self-maintaining and instrumental activities of daily living. Gerontologist. 1969;9:179–86. 5349366

[45] Galvin JE, Roe CM, Coats MA, Morris JC. Patient’s rating of cognitive ability: using the AD8, a brief informant interview, as a self-rating tool to detect dementia. Arch Neurol. 2007;64:725–30. 10.1001/archneur.64.5.725. 17502472 10.1001/archneur.64.5.725

[46] Burke WJ, Roccaforte WH, Wengel SP. The Short Form of the Geriatric Depression Scale: a comparison with the 30-item form. J Geriatr Psychiatry Neurol. 1991;4:173–8. 10.1177/089198879100400310. 1953971 10.1177/089198879100400310

[47] Russell D, Peplau LA, Cutrona CE. The revised UCLA Loneliness Scale: concurrent and discriminant validity evidence. J Pers Soc Psychol. 1980;39:472–80. 10.1037/0022-3514.39.3.472. 7431205 10.1037//0022-3514.39.3.472

[48] Cohen S, Kamarck T, Mermelstein R. A global measure of perceived stress. J Health Soc Behav. 1983;24:385–96. 6668417

[49] Lubben J, Blozik E, Gillmann G, Iliffe S, von Renteln KW, Beck JC, et al. Performance of an abbreviated version of the Lubben Social Network Scale among three European community-dwelling older adult populations. Gerontologist. 2006;46:503–13. 10.1093/geront/46.4.503. 16921004 10.1093/geront/46.4.503

[50] Yan C-G, Wang X-D, Zuo X-N, Zang Y-F. DPABI: data processing & analysis for (Resting-State) brain imaging. Neuroinformatics. 2016;14:339–51. 10.1007/s12021-016-9299-4. 27075850 10.1007/s12021-016-9299-4

[51] Tournier J-D, Smith R, Raffelt D, Tabbara R, Dhollander T, Pietsch M, et al. MRtrix3: A fast, flexible and open software framework for medical image processing and visualisation. Neuroimage. 2019;202: 116137. 10.1016/j.neuroimage.2019.116137. 31473352 10.1016/j.neuroimage.2019.116137

[52] Fischl B. FreeSurfer. NeuroImage. 2012;62:774–81. 10.1016/j.neuroimage.2012.01.021. 22248573 10.1016/j.neuroimage.2012.01.021 PMC3685476

[53] Fan L, Li H, Zhuo J, Zhang Y, Wang J, Chen L, et al. The Human Brainnetome Atlas: a new brain Atlas based on connectional architecture. Cereb Cortex. 2016;26:3508–26. 10.1093/cercor/bhw157. 27230218 10.1093/cercor/bhw157 PMC4961028

[54] Wang J, Wang X, Xia M, Liao X, Evans A, He Y. GRETNA: a graph theoretical network analysis toolbox for imaging connectomics. Front Hum Neurosci. 2015;9:1–16. 10.3389/fnhum.2015.00386. 26175682 10.3389/fnhum.2015.00386 PMC4485071

[55] Hagmann P, Cammoun L, Gigandet X, Meuli R, Honey CJ, Van Wedeen J, et al. Mapping the structural core of human cerebral cortex. PLoS Biol. 2008;6:1479–93. 10.1371/journal.pbio.0060159. 10.1371/journal.pbio.0060159 PMC2443193 18597554

[56] Zhang J, Cheng W, Liu Z, Zhang K, Lei X, Yao Y, et al. Neural, electrophysiological and anatomical basis of brain- network variability and its characteristic changes in mental disorders. Brain. 2016;139:2307–21. 10.1093/aww143. 27421791 10.1093/brain/aww143

[57] Johansen-Berg H, Rushworth MFS. Using diffusion imaging to study human connectional anatomy. Annu Rev Neurosci. 2009;32:75–94. 10.1146/annurev.neuro.051508.135735. 19400718 10.1146/annurev.neuro.051508.135735

[58] van den Heuvel MP, Hulshoff Pol HE. Exploring the brain network: a review on resting-state fMRI functional connectivity. Eur Neuropsychopharmacol. 2010;20:519–34. 10.1016/j.euroneuro.2010.03.008. 20471808 10.1016/j.euroneuro.2010.03.008

[59] Purves D, editor. Neuroscience. 3rd ed. Sunderland, Mass: Sinauer Associates, Publishers; 2004.

[60] Pedregosa F, Varoquaux G, Gramfort A, Michel V, Thirion B, Grisel O, et al. Scikit-learn: machine learning in Python. J Machine Learning Res. 2011;12:2825–30.

[61] Shen X, Finn ES, Scheinost D, Rosenberg MD, Chun MM, Papademetris X, et al. Using connectome-based predictive modeling to predict individual behavior from brain connectivity. Nat Protoc. 2017;12:506–18. 10.1038/nprot.2016.178. 28182017 10.1038/nprot.2016.178 PMC5526681

[62] Sporns O. Graph theory methods: applications in brain networks 2018:111–20. 10.31887/DCNS.2018.20.2/osporns. 10.31887/DCNS.2018.20.2/osporns PMC6136126 30250388

[63] Conrod PJ, Castellanos-Ryan N, Mackie C. Long-term effects of a personality-targeted intervention to reduce alcohol use in adolescents. J Consult Clin Psychol. 2011;79:296–306. 10.1037/a0022997. 21500886 10.1037/a0022997

[64] Jack CR, Bernstein MA, Fox NC, Thompson P, Alexander G, Harvey D, et al. The Alzheimer’s Disease Neuroimaging Initiative (ADNI): MRI methods. J Magn Reson Imaging. 2008;27:685–91. 10.1002/jmri.21049. 18302232 10.1002/jmri.21049 PMC2544629

[65] Stam CJ, Van Dijk BW. Synchronization likelihood: an unbiased measure of generalized synchronization in multivariate data sets. Phys Nonlinear Phenom. 2002;163:236–51. 10.1016/S0167-2789(01)00386-4.

[66] Racz FS, Stylianou O, Mukli P, Eke A. Multifractal and entropy-based analysis of delta band neural activity reveals altered functional connectivity dynamics in schizophrenia. Front Syst Neurosci. 2020;14:49. 10.3389/fnsys.2020.00049. 32792917 10.3389/fnsys.2020.00049 PMC7394222

[67] Buckner RL, Bandettini PA, O’Craven KM, Savoy RL, Petersen SE, Raichle ME, et al. Detection of cortical activation during averaged single trials of a cognitive task using functional magnetic resonance imaging. Proc Natl Acad Sci. 1996;93:14878–83. 10.1073/pnas.93.25.14878. 8962149 10.1073/pnas.93.25.14878 PMC26230

[68] Cabral J, Kringelbach ML, Deco G. Functional connectivity dynamically evolves on multiple time-scales over a static structural connectome: models and mechanisms. Neuroimage. 2017;160:84–96. 10.1016/j.neuroimage.2017.03.045. 28343985 10.1016/j.neuroimage.2017.03.045

[69] Sherry A, Henson RK. Conducting and interpreting canonical correlation analysis in personality research: a user-friendly primer. J Pers Assess. 2005;84:37–48. 10.1207/s15327752jpa8401_09. 15639766 10.1207/s15327752jpa8401_09

[70] Li L, Li LMW, Ma J, Lu A, Dai Z. The relationship between personality traits and well-being via brain functional connectivity. J Happiness Stud. 2023;24:2127–52. 10.1007/s10902-023-00674-y.

[71] Cole JH, Poudel RPK, Tsagkrasoulis D, Caan MWA, Steves C, Spector TD, et al. Predicting brain age with deep learning from raw imaging data results in a reliable and heritable biomarker. Neuroimage. 2017;163:115–24. 10.1016/j.neuroimage.2017.07.059. 28765056 10.1016/j.neuroimage.2017.07.059

[72] Antonenko D, Brauer J, Meinzer M, Fengler A, Kerti L, Friederici AD, et al. Functional and structural syntax networks in aging. Neuroimage. 2013;83:513–23. 10.1016/j.neuroimage.2013.07.018. 23867559 10.1016/j.neuroimage.2013.07.018

[73] Puxeddu MG, Faskowitz J, Betzel RF, Petti M, Astolfi L, Sporns O. The modular organization of brain cortical connectivity across the human lifespan. NeuroImage 2020;218. 10.1016/j.neuroimage.2020.116974. 10.1016/j.neuroimage.2020.116974 32450249

[74] Bastin ME, Piatkowski JP, Storkey AJ, Brown LJ, MacLullich AMJ, Clayden JD. Tract shape modelling provides evidence of topological change in corpus callosum genu during normal ageing. Neuroimage. 2008;43:20–8. 10.1016/j.neuroimage.2008.06.047. 18687404 10.1016/j.neuroimage.2008.06.047

[75] Raz N, Ghisletta P, Rodrigue KM, Kennedy KM, Lindenberger U. Trajectories of brain aging in middle-aged and older adults: regional and individual differences. Neuroimage. 2010;51:501–11. 10.1016/j.neuroimage.2010.03.020. 20298790 10.1016/j.neuroimage.2010.03.020 PMC2879584

[76] Chen Q, Xia Y, Zhuang K, Wu X, Liu G, Qiu J. Decreased inter-hemispheric interactions but increased intra-hemispheric integration during typical aging. Aging. 2019;11:10100–15. 10.18632/aging.102421. 31761785 10.18632/aging.102421 PMC6914428

[77] Delvenne J-F, Castronovo J. Reduced inter-hemispheric interference in ageing: evidence from a divided field Stroop paradigm. Brain Cogn. 2018;122:26–33. 10.1016/j.bandc.2018.01.008. 29407788 10.1016/j.bandc.2018.01.008

[78] Mather M. The affective neuroscience of aging. Annu Rev Psychol. 2016;67:213–38. 10.1146/annurev-psych-122414-033540. 26436717 10.1146/annurev-psych-122414-033540 PMC5780182

[79] Dadario NB, Sughrue ME. The functional role of the precuneus. Brain. 2023;146:3598–607. 10.1093/brain/awad181. 37254740 10.1093/brain/awad181

[80] Freeman LC. A set of measures of centrality based on betweenness. Sociometry. 1977;40:35–41. 10.2307/3033543.

[81] Watts DJ, Strogatz SH. Collective dynamics of ‘small-world’ networks. Nature. 1998;393:440–2. 10.1038/30918. 9623998 10.1038/30918

[82] Culham JC, Kanwisher NG. Neuroimaging of cognitive functions in human parietal cortex. Curr Opin Neurobiol. 2001;11:157–63. 10.1016/S0959-4388(00)00191-4. 11301234 10.1016/s0959-4388(00)00191-4

[83] Palejwala AH, O’Connor KP, Pelargos P, Briggs RG, Milton CK, Conner AK, et al. Anatomy and white matter connections of the lateral occipital cortex. Surg Radiol Anat. 2020;42:315–28. 10.1007/s00276-019-02371-z. 31734739 10.1007/s00276-019-02371-z

[84] Malach R, Reppas JB, Benson RR, Kwong KK, Jiang H, Kennedy WA, et al. Object-related activity revealed by functional magnetic resonance imaging in human occipital cortex. Proc Natl Acad Sci. 1995;92:8135–9. 10.1073/pnas.92.18.8135. 7667258 10.1073/pnas.92.18.8135 PMC41110

[85] Vincent JL, Kahn I, Snyder AZ, Raichle ME, Buckner RL. Evidence for a frontoparietal control system revealed by intrinsic functional connectivity. J Neurophysiol. 2008;100:3328–42. 10.1152/jn.90355.2008. 18799601 10.1152/jn.90355.2008 PMC2604839

[86] Kramer AF, Humphrey DG, Larish JF, Logan GD. Aging and inhibition: beyond a unitary view of inhibitory processing in attention. Psychol Aging. 1994;9:491–512. 10.1037/0882-7974.9.4.491. 7893421

[87] Briggs RG, Lin Y-H, Dadario NB, Kim SJ, Young IM, Bai MY, et al. Anatomy and white matter connections of the middle frontal gyrus. World Neurosurg. 2021;150:e520–9. 10.1016/j.wneu.2021.03.045. 33744423 10.1016/j.wneu.2021.03.045

[88] Gutchess AH, Welsh RC, Hedden T, Bangert A, Minear M, Liu LL, et al. Aging and the neural correlates of successful picture encoding: frontal activations compensate for decreased medial-temporal activity. J Cogn Neurosci. 2005;17:84–96. 10.1162/0898929052880048. 15701241 10.1162/0898929052880048

[89] Cabeza R, Dennis NA. Frontal lobes and aging. Princ Front Lobe Funct 2013:628–52. 10.1093/med/9780199837755.003.0044.

[90] Ferrucci L, Fabbri E. Inflammageing: chronic inflammation in ageing, cardiovascular disease, and frailty. Nat Rev Cardiol. 2018;15:505–22. 10.1038/s41569-018-0064-2. 30065258 10.1038/s41569-018-0064-2 PMC6146930

[91] Fan F, Liao X, Lei T, Zhao T, Xia M, Men W, et al. Development of the default-mode network during childhood and adolescence: a longitudinal resting-state fMRI study. NeuroImage 2021;226. 10.1016/j.neuroimage.2020.117581. 10.1016/j.neuroimage.2020.117581 33221440

[92] Hampson M, Tokoglu F, Sun Z, Schafer RJ, Skudlarski P, Gore JC, et al. Connectivity-behavior analysis reveals that functional connectivity between left BA39 and Broca’s area varies with reading ability. NeuroImage 2006;31:513–9. 10/fktnpm. 10.1016/j.neuroimage.2005.12.040 16497520

[93] Liu J, Xia M, Dai Z, Wang X, Liao X, Bi Y, et al. Intrinsic brain hub connectivity underlies individual differences in spatial working memory. Cereb Cortex. 2017;27:5496–508. 10.1093/cercor/bhw317. 28334075 10.1093/cercor/bhw317

[94] Lee J, Burkett BJ, Min HK, Senjem ML, Lundt ES, Botha H, et al. Deep learning-based brain age prediction in normal aging and dementia. Nat Aging. 2022;2:412–24. 10.1038/s43587-022-00219-7. 37118071 10.1038/s43587-022-00219-7 PMC10154042

[95] Troy AS, Willroth EC, Shallcross AJ, Giuliani NR, Gross JJ, Mauss IB. Psychological resilience: an affect-regulation framework. Annu Rev Psychol. 2023;74:547–76. 10.1146/annurev-psych-020122-041854. 36103999 10.1146/annurev-psych-020122-041854 PMC12009612

[96] Bernier PJ, Gourdeau C, Carmichael P-H, Beauchemin J-P, Voyer P, Hudon C, et al. It’s all about cognitive trajectory: accuracy of the cognitive charts–MoCA in normal aging, MCI, and dementia. J Am Geriatr Soc 2023;71:214–20. 10/gsgvdm. 10.1111/jgs.18029 PMC9870845 36102601

