# Supplementary material for: The negative relationship between brain-age gap and psychological resilience defines the age-related neurocognitive status in older people
Source: GeroScience. 2025 Jan 28;47(3):4023–40. doi: 10.1007/s11357-025-01515-x (PMC12181537; doi:10.1007/s11357-025-01515-x)
Supplement: Supplementary file 1 — Supplementary file1 (DOCX 2803 KB) [file 11357_2025_1515_MOESM1_ESM.docx]

**Supplementary Material**

***Validation Results***

In our evaluation of the brain-age prediction model using a separate dataset from the Alzheimer's Disease Neuroimaging Initiative (ADNI) cohort, we observed a MAE of 8.876 and a RMSE of 11.258, which were not as favorable as the results obtained in our primary study. Although this model did not perform as well as in our main study, there remains a correlation between chronological age and predicted brain age (*r* = 0.361, *p* < 0,001, **Figure S1**). One potential explanation for this underperformance could be the differences in the demographic characteristics of the datasets. Specifically, the training dataset was sourced from a population in China, while the ADNI dataset consists of subjects mostly from Canada and America. This geographical discrepancy may introduce variability in the data, affecting the model's ability to generalize across populations. Additionally, there were inconsistencies in the age range of the subjects in the two datasets, which could further contribute to the observed performance issues.

While our brain-age prediction model utilizing dynamic features derived from SL achieved a MAE of 4.244 and a RMSE of 5.319, with the hyperparameter alpha optimized at 3.875, its performance was not as favorable as that reported in the main text. Among the 428 features selected for analysis, it is noteworthy that none from SL, while all were derived from SC. We present the top 20 features with the highest weights identified in our analysis in **Figure S2**, underscoring the predominance of SC-derived features in our predictive model. Among all selected features, the SC between left inferior parietal lobule and left middle temporal gyrus exhibits the highest weight at 0.184, followed by SC between right thalamus with left middle temporal gyrus at -0.139. This suggests that while exploring dynamic features is valuable, the current model remains robust with the SC-derived features, warranting its retention in future investigations.


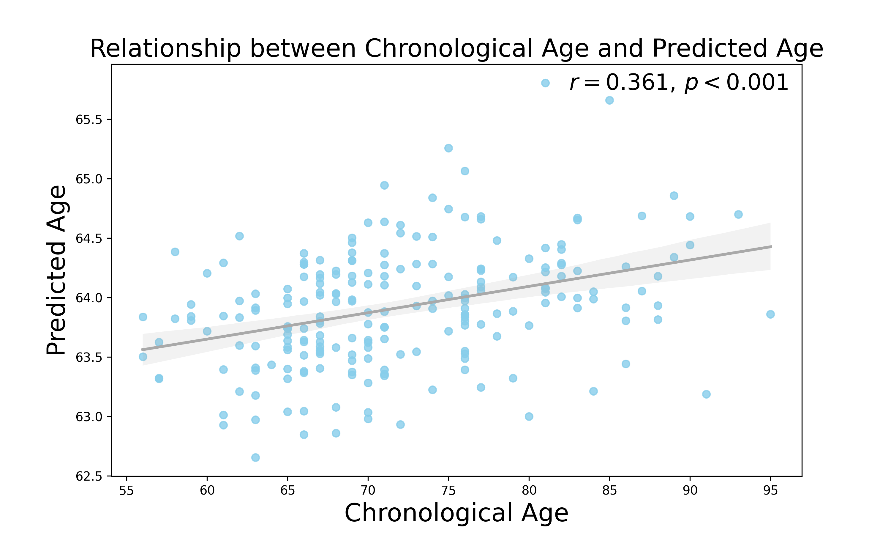


**Figure S1** Relationship between chronological age and predicted age in from the original brain-age prediction model in the ADNI dataset. The regression line indicates a significant positive relationship, with a correlation coefficient 𝑟 = 0.361 and *p* < 0.001. Each node represents an individual subject. The shaded area represents the 95% confidence interval for the predictions.


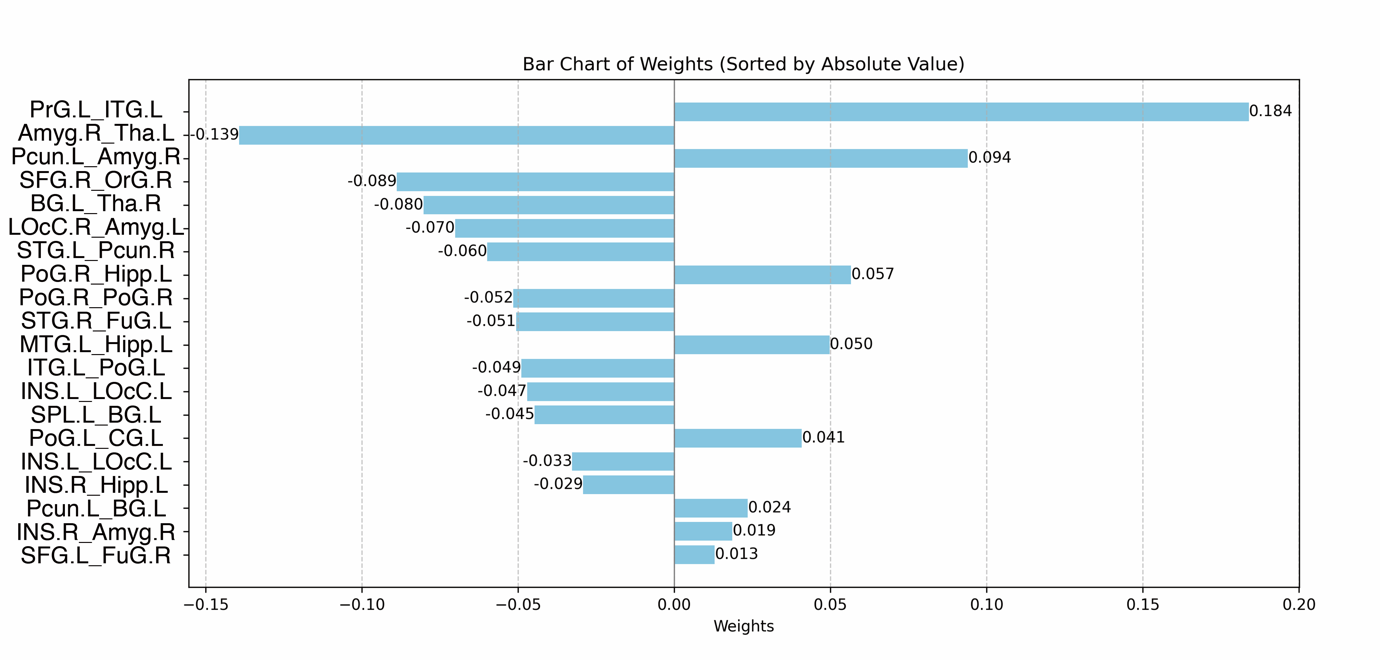


**Figure S2** Top 20 features with the highest weights identified in the brain age prediction model using the Synchronization Likelihood (SL) method for dynamic feature calculation. The abbreviations used here include SFG (Superior Frontal Gyrus), MFG (Middle Frontal Gyrus), IFG (Inferior Frontal Gyrus), OrG (Orbital Gyrus), PrG (Precentral Gyrus), SPL (Superior Parietal Lobule), STG (Superior Temporal Gyrus), MTG (Middle Temporal Gyrus), ITG (Inferior Temporal Gyrus), FuG (Fusiform Gyrus), Amyg (Amygdala), Hipp (Hippocampus), BG (Basal Ganglia), Tha (Thalamus), INS (Insular Gyrus), and Pcun (Precuneus). The notation 'R' indicates the right hemisphere, while 'L' signifies the left hemisphere.

**Table S1** The significant corelations between brain-age gap and nodal characteristics within the functional network (*p* < 0.05). Asterisks indicate FDR-corrected significance at *p* = 0.05.

**Betweenness centrality**

| **Region** | ***rho*** | ***P value*** |
| --- | --- | --- |
| Right Middle Frontal Gyrus | 0.221963 | 0.035501 |
| Left Orbital Gyrus | 0.237133 | 0.024424 |
| Left Precentral Gyrus | -0.22343 | 0.034274 |
| Right Precentral Gyrus | 0.30904 | 0.003039 |
| Left Superior Temporal Gyrus | -0.22025 | 0.036983 |
| Left Inferior Temporal Gyrus | -0.21199 | 0.04487 |
| Left Parahippocampal Gyrus | 0.234594 | 0.026041 |
| Left Inferior Parietal Lobule | -0.21609 | 0.040801 |
| Left Postcentral Gyrus | 0.237924 | 0.023938 |
| Left Cingulate Gyrus | -0.21826 | 0.038765 |
| Left MedioVentral Occipital Cortex | 0.256707 | 0.014591 |
| Left lateral Occipital Cortex | -0.21592 | 0.040955 |
| Right Basal Ganglia | -0.25486 | 0.015344 |
| Right Thalamus | 0.21007 | 0.046892 |

**Degree centrality**

| **Region** | ***rho*** | ***p value*** |
| --- | --- | --- |
| Right Middle Frontal Gyrus | 0.223033 | 0.034601 |
| Right Orbital Gyrus | 0.246166 | 0.019345 |
| Right Precentral Gyrus | 0.334208 | 0.001285 |
| Left Superior Temporal Gyrus | -0.215 | 0.041846 |
| Left Superior Temporal Gyrus | -0.2089 | 0.048155 |
| Left Superior Parietal Lobule | -0.21247 | 0.044378 |
| Left Inferior Parietal Lobule | -0.20913 | 0.047911 |
| Left Postcentral Gyrus | -0.21065 | 0.04627 |
| Left lateral Occipital Cortex | -0.22608 | 0.032144 |

**Clustering coefficient**

| **Region** | ***rho*** | ***p value*** |
| --- | --- | --- |
| Right Superior Frontal Gyrus | 0.316117 | 0.002403 |
| Left Superior Temporal Gyrus | 0.284999 | 0.006476 |
| Right Middle Temporal Gyrus | -0.25711 | 0.014433 |
| Right Parahippocampal Gyrus | 0.275824 | 0.008504 |
| Left Insular Gyrus | -0.24495 | 0.019971 |
| Left MedioVentral Occipital Cortex | -0.22845 | 0.030334 |

**Efficiency**

| **Region** | ***rho*** | ***p value*** |
| --- | --- | --- |
| Right Middle Frontal Gyrus | 0.23285 | 0.027203 |
| Right Precentral Gyrus | 0.397677* | 0.000104* |
| Left Superior Temporal Gyrus | -0.22364 | 0.0341 |
| Right Cingulate Gyrus | 0.236906 | 0.024565 |

**Table S2** The significant corelations between brain-age gap and nodal characteristics within the structural network (*p* < 0.05).

**Betweenness centrality**

| **Region** | ***rho*** | ***p value*** |
| --- | --- | --- |
| Right Middle Temporal Gyrus | -0.2943 | 0.00487 |
| Right Inferior Temporal Gyrus | 0.27194 | 0.00952 |
| Left Parahippocampal Gyrus | 0.21601 | 0.04087 |
| Right Parahippocampal Gyrus | 0.26362 | 0.01206 |
| Right Parahippocampal Gyrus | 0.2295 | 0.02956 |
| Left Posterior Superior Temporal Sulcus | 0.30307 | 0.00369 |
| Right Posterior Superior Temporal Sulcus | 0.22399 | 0.03382 |
| Right Inferior Parietal Lobule | -0.2164 | 0.04047 |
| Right Insular Gyrus | -0.2406 | 0.02233 |
| Right Cingulate Gyrus | -0.2397 | 0.02287 |
| Left Cingulate Gyrus | 0.35168 | 0.00068 |
| Right Hippocampus | -0.2083 | 0.04887 |
| Left Basal Ganglia | 0.24048 | 0.02242 |

**Degree centrality**

| **Region** | ***rho*** | ***p value*** |
| --- | --- | --- |
| Left Middle Frontal Gyrus | 0.24365 | 0.02066 |
| Right Inferior Frontal Gyrus | -0.2296 | 0.02951 |
| Left Fusiform Gyrus | -0.209 | 0.04807 |
| Left posterior Superior Temporal Sulcus | 0.22874 | 0.03012 |
| Left Cingulate Gyrus | 0.25786 | 0.01414 |
| Right Cingulate Gyrus | 0.2184 | 0.03864 |
| Left lateral Occipital Cortex | -0.2201 | 0.03712 |
| Left Basal Ganglia | 0.21621 | 0.04068 |

**Clustering coefficient**

| **Region** | ***rho*** | ***p value*** |  |
| --- | --- | --- | --- |
| Right Superior Frontal Gyrus | -0.2794 | 0.00765 | |
| Right Orbital Gyrus | -0.2574 | 0.01431 | |
| Left Orbital Gyrus | 0.22826 | 0.03047 | |
| Left Paracentral Lobule | 0.23632 | 0.02493 | |
| Right Paracentral Lobule | 0.21364 | 0.0432 | |
| Right Middle Temporal Gyrus | 0.24141 | 0.02189 | |
| Left Inferior Temporal Gyrus | 0.22633 | 0.03195 | |
| Right Parahippocampal Gyrus | -0.2565 | 0.01466 | |
| Left posterior Superior Temporal Sulcus | -0.2102 | 0.04675 | |
| Left Precuneus | 0.24506 | 0.01991 | |
| Right Insular Gyrus | 0.24976 | 0.0176 | |
| Right Cingulate Gyrus | 0.21031 | 0.04664 | |
| Left Cingulate Gyrus | 0.36332 | 0.00043 | |
| Left Cingulate Gyrus | 0.35857 | 0.00052 | |
| Right MedioVentral Occipital Cortex | -0.2928 | 0.0051 | |

**Efficiency**

| **Region** | ***rho*** | ***p value*** | |  |
| --- | --- | --- | --- | --- |
| Right Middle Frontal Gyrus | 0.21182 | | 0.04505 | |
| Right Inferior Frontal Gyrus | -0.2163 | | 0.0406 | |
| Right Orbital Gyrus | -0.2195 | | 0.03761 | |
| Right Middle Temporal Gyrus | -0.2502 | | 0.01741 | |
| Left posterior Superior Temporal Sulcus | 0.30145 | | 0.00389 | |
| Left Cingulate Gyrus | 0.23432 | | 0.02622 | |
| Right Cingulate Gyrus | 0.24293 | | 0.02105 | |
| Left Hippocampus | 0.2128 | | 0.04404 | |
